# Deep analysis and optimization of CARD antibiotic resistance gene discovery models

**DOI:** 10.1186/s12864-019-6318-5

**Published:** 2019-12-30

**Authors:** Haobin Yao, Siu-Ming Yiu

**Affiliations:** Department of Computer Science, The University of Hong Kong, Pok Fu Lam, Hong Kong, SAR China

**Keywords:** Antibiotic resistance gene, CARD database, RND efflux pumps

## Abstract

**Background:**

Identification of antibiotic resistance genes from environmental samples has been a critical sub-domain of gene discovery which is directly connected to human health. However, it is drawing extraordinary attention in recent years and regarded as a severe threat to human health by many institutions around the world. To satisfy the needs for efficient ARG discovery, a series of online antibiotic resistance gene databases have been published. This article will conduct an in-depth analysis of CARD, one of the most widely used ARG databases.

**Results:**

The decision model of CARD is based the alignment score with a single ARG type. We discover the occasions where the model is likely to make false prediction, and then propose an optimization method on top of the current CARD model. The optimization is expected to raise the coherence with BLAST homology relationships and improve the confidence for identification of ARGs using the database.

**Conclusions:**

The absence of public recognized benchmark makes it challenging to evaluate the performance of ARG identification. However, possible wrong predictions and methods for resolving the problem can be inferred by computational analysis of the identification method and the underlying reference sequences. We hope our work can bring insight to the mission of precise ARG type classifications.

## Background

In recent years, the emergence of antibiotic resistance is accelerating across the world [1]. A wide spectrum of antibiotics which have saved millions of lives since the 1950s are getting less effective in the treatment of bacterial infections [2], arousing serious attention of medical researchers and public health institutions over the world [3]. The major factors that account for the spread of resistant bacteria are recognized to be the unrestricted use of antibiotic drugs for the treatment of both human and animal diseases, combined with the insufficient efficiency of new drug development [1]. Nonetheless, fast and reliable analysis of genes that cause the resistance to certain drugs is the prerequisite to carry out further steps to design and build solutions. Fortunately, at the same time genome sequencing technology and dedicated bioinformatics software are also evolving rapidly, boosting our ability to deal with the deepening crisis [4].

To satisfy the needs of ARG detection for researchers and medical institutions, a series of antibiotic resistance gene databases have been published online, such as ARDB [5], CARD [6], SARG [7, 8], and NCBI-AMRFinder (https://www.ncbi.nlm.nih.gov/pathogens/antimicrobial-resistance/AMRFinder/). These databases provide a public platform for efficient computational analysis and collaborative researches [4].

The ARDB [5], a classical comprehensive database that contains over 1000 genes with annotations of their ARG types, has been used in a lot of applications. It’s now no longer maintained and mainly replaced by The Comprehensive Antibiotic Resistance Database (CARD) [6]. Initially online in 2015 and expanded in 2016, CARD now has over 2500 ARG entries with a monthly update. Each entry represents a type of ARG like mcr-1 [9, 10], mcr-2 [11], etc. And these entries are placed in a hierarchical structure of gene ontology terms which are compatible with the system published by the GO consortium (http://geneontology.org/). For each entry, CARD provides both DNA and protein representative sequences and a bit score threshold to report ARG hits by BLAST alignment. Also, CARD collects over 140,000 sequences from NCBI and classifies them to generate the prevalence data of ARG types in the environment. In later parts of the article, we will use these prevalence sequences to study CARD in detail.

SARG is a more recent ARG databases published in 2016 [7] and expanded in 2018 [8]. Based on ARDB and CARD, it now contains more than 12,000 protein sequences, organized into 1208 categories of ARGs. The categories of sequences are decided by keyword-searching in the ARG type annotations of ARGB and CARD, combined with similarity search of classified sequences in the NCBI-NR database [9]. Its open-source ARG discovery pipeline will let users set BLAST e-value and identity threshold as parameters (https://github.com/biofuture/Ublastx_stageone).

Another novel ARG database, AMRFinder (NCBI project ID: PRJNA313047), is developed by NCBI bases on CARD. It contains significantly more sequences than CARD (totally over 4000), but additional sequences mostly show high similarity to existing sequences in CARD). An important feature of AMRFinder is the adoption of Hidden-Markov Models (HMM) instead of BLAST alignment. HMM models are constructed for each family of antibiotic resistance genes. And then TC cut-offs are trained with proteins catalogs such as ResFam [12] and PFam [13], where protein families are collected with functional annotations.

Despite the progress on building ARG databases, the lack of universally accepted benchmark hinders the validation of query precision and integration across different references and methods. A previous review [14] that evaluate ARG databases with a small number of known ARG sequences indicates that CARD reports the most number of correct predictions.

The ARG type annotations in these databases are mainly collected from past literatures, and approaches of different databases to report a type of ARG are largely different. In the ideal situation, we hope to have a “golden standard” benchmark that contains test sequences and their reliable ARG type information. However, such universal accepted benchmark is still not available, which makes validation of query precision and integration of different ARG databases remain challenging missions.

However, we can still find insights on potential false cases by dedicated analysis of the specific methods adopted by a certain database. Here we will conduct an in-depth inspection of the decision model used by CARD database. Not only the computational methods are described, but also the effects of trained models on certain query data will be analyzed. In result, we spot occasions where the database is likely to make false prediction. Moreover, we will formally describe ambiguous cases due to the logic of the ARG type decision process which merely tests whether a query sequence is sufficiently similar to a single ARG type. After locating and defining the problem, we propose an optimization method on top of current CARD models. The optimization is expected to make the models more coherent with BLAST homology relationships and reduce the expected error rate.

## Methods

### Inconsistency between CARD models and BLAST homology

To discover ARGs from query DNA sequences, CARD predicts Open Reading Frames from query data using Prodigal [15], and then performs protein-protein alignment with BLASTP [16]. A critical feature of CARD is that it provides a trained BLASTP alignment bit-score threshold for each type of antibiotic resistance gene. In contrast, other existing databases mainly use an empirical or user-set parameter for the discovery of all genes. For example, another popular database Resfinder [17] requires percent-identity and coverage on reference genes as input parameters. The reason the approach of CARD is more appropriate is that ARGs in one category may be almost identical to each other while some categories can contain ARGs with relatively low similarity. We take two types of ARGs which are represented both CARD and Resfinder for illustration – tet(A) [18] and mcr-1 [9, 10]. When all sequences of tet(A) in Resfinder are aligned to sequences of the same type in CARD, the mean percent identity is 99.6%. However, for mcr-1 the mean percent identity is 47.2%. The degree of similarity inside a type of ARG could be very different, therefore it’s more reasonable to have a specific threshold for a specific type of ARGs. However, the flexible models could give type classifications that are not coherent with BLAST alignment homology relationships. Since the model only considers whether the bit score passes the threshold of a single ARG type, it can happen that the type classification of a query sequence is not the best BLAST hit. For example, if ARG type A reports higher bit score than type B for a given query sequence, but the pre-trained threshold of A is much higher than B, then type B could be chosen instead of A. Since BLAST alignment serves as a generally accepted method to evaluate the similarity between genome sequences, we consider the occurrences of incoherence to BLAST homology to be ambiguous cases that need special attention.

### Ambiguity in RND efflux pumps

RND efflux pumps [19] are a superfamily of transporters that have garnered intensive research efforts. Studies have revealed that they play critical roles in the development of multidrug resistance in various kinds of bacteria. In CARD databases, a series of ARG types in this family are presented. We notice that one gene (adeF) in this family is given a relatively low threshold – bit score 750 which allows sequences lower than 50% identity to be reported as an instance of this type. However, other genes mainly require much higher identity. MexF, another ARG in RND family, requires bit score 2200, which only allows almost totally identical sequence to be reported. Since genes in the RND family can display a certain level of homology even though they belong to different sub-types, ambiguous cases described in the last section are likely to occur. This can be clearly demonstrated with the help of ARG sequences in SARG [7], another ARG databases that contain ARG protein sequences in the RND family. There are over 300 protein sequences with MexF annotation in SARG. We align these sequences to CARD databases. In result, the MexF entry in CARD is certainly the best BLAST alignment hits for these sequences. However, they will be classified to adeF under the curated model of CARD since their bit score does not reach the threshold of MexF. Instead, their bit score to the adeF entry exceeds the threshold of the ARG type so that MexF sequences in SARG are all classified to the adeF entry by the CARD model.

### Describe ambiguity in CARD database

To describe and quantify the ambiguity inside the classification model of CARD in a systematic manner, we define FN-ambiguity and Coherence-ratio.

First of all, we have several basic variables:

1. N_i_ = the number of prevalence sequences that can align to ARO entry A_i_
2. C_i_ = the number of sequences that are currently classified to entry A_i_

Then we define two indicators with the pre-trained bit-score cut-off. One is potential False-Negatives for some ARO entries, which we would like to reduce, and Coherence-ratio with respective to BLAST best-hits which we intend to maximize.

A) *FN-ambiguity:*

If a prevalence sequence S_i_ not annotated to the ARG A_j_ has (bit-score, percent identity) both larger than another sequence S_k_ which is annotated to the ARG, then S_i_ is potential FN for this ARO. Let M_j_ = the number of such potential FN sequences, we have:

1 $$ F{N}_{ratio\left({A}_j\right)}={M}_j/{N}_j $$

Also, we say that each such (S_i_, S_j_) is an *FN-ambiguous* pair for ARO A_j_.

For each potential FP sequence S_i_ respect to ARO entry A_j_, K_i_ = the number of sequences with lower (bit-score, identity) than S_i_ and annotated to G_j._ We can calculate the probability of the occurrence of *FN-ambiguous* for an ARO A_j_ by:

2 $$ P\mathrm{FN}-\mathrm{ambiguous}\ \mathrm{pair}=\frac{\sum Ki,j}{\left( Nj- Cj\right)\ast Cj} $$

In the worst case, each of the sequences not annotated to the entry (N-C) has (bit-score, identity) larger than all sequences annotated to the entry (C), then *P* = 1.

**Fig. 1:**
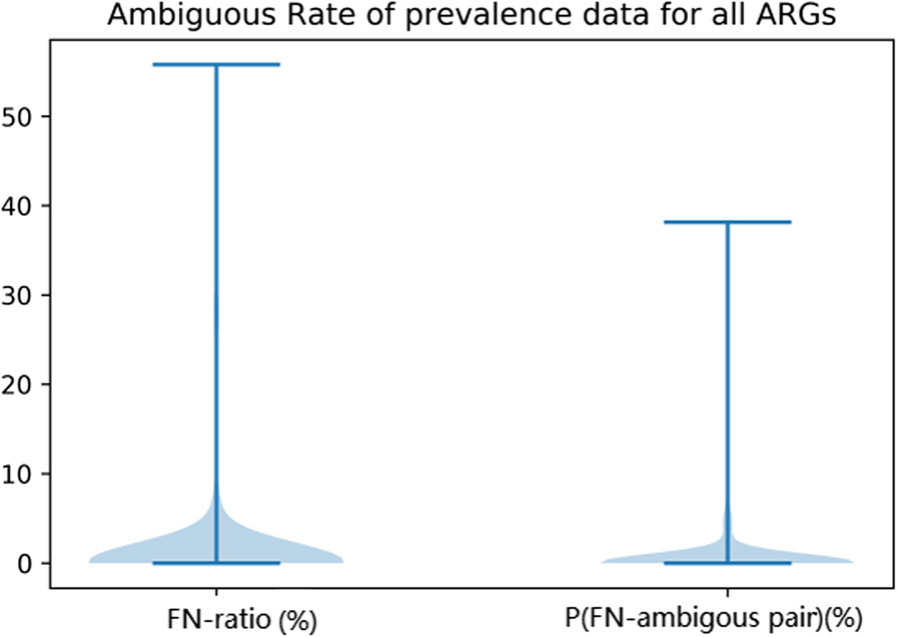
Ambiguity for all ARGs in CARD

**Fig. 2:**
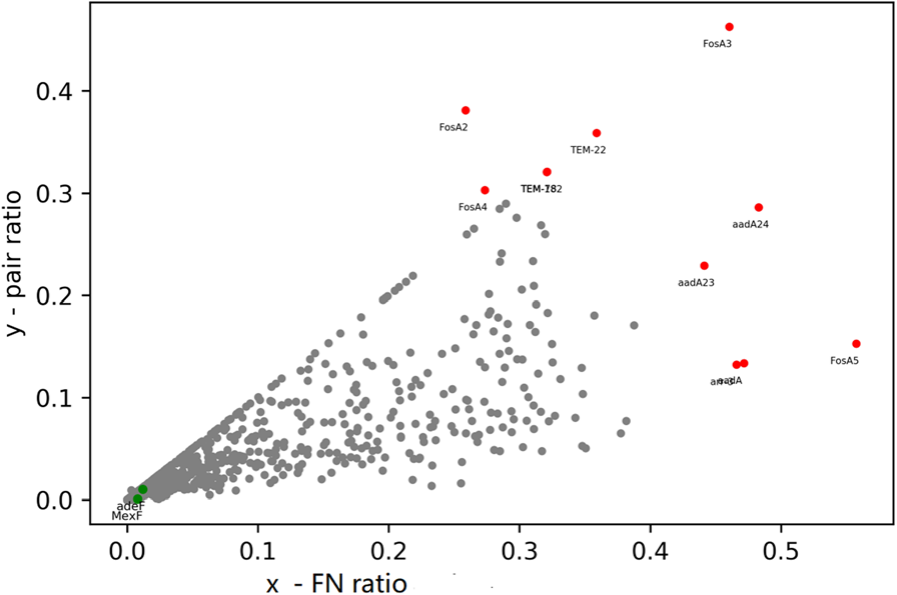
Exceptional ARO entries with high-ambiguity ratios

In the above example of MexF the FN-ratio is 0.79%, with P_FN-ambiguous pair_ at 0.07%. Over the whole database, the mean (sequence-ambiguity-ratio, pair-ambiguity-ratio) is (3.1, 1.6%). We can see in Fig. 1 that both ratios gather below 5% with a smaller number of exceptions. Ratio coordinates of MexF, adeF, and entries with exceptionally high ratios are shown in Fig. 2.

B) *Coherence Ratio:*

For a prevalence sequence S_i_, suppose its best-hit ARO entry Ai (the entry with the highest BLASTP alignment bit-score. If current ARG type annotation of Si is also Ai, we say S_i_ is a coherent instance for A_i_. Let the number of coherent instances for A_i_ be TP_i_, and the total number of sequences with A_j_ as the best-hit ARO be B_j_, we define:

3 $$ Coherence\ \left({A}_j\right)=T{P}_j/{B}_j $$

**Fig. 3:**
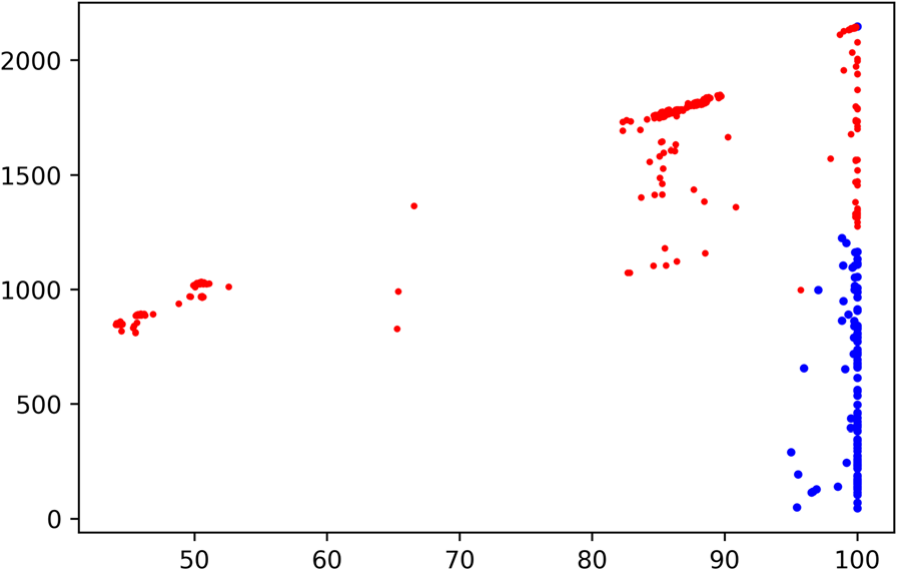
Problem set of MexF

Since BLAST is the most well-established software to measure the homology between sequences, it’s reasonable to evaluate the coherence of the homology relationships given by CARD ARG models and BLASP alignment. We see that in many occasions that the ARG type annotation of the prevalence sequence is not its best-hit ARO entry. Take MexF (ARO: 3000804) mentioned in previous experiments as an example (Fig. 3). We see a large portion of prevalence sequences with MexF as their best-hits but annotated to adeF (red points in figure).

**Fig. 4:**
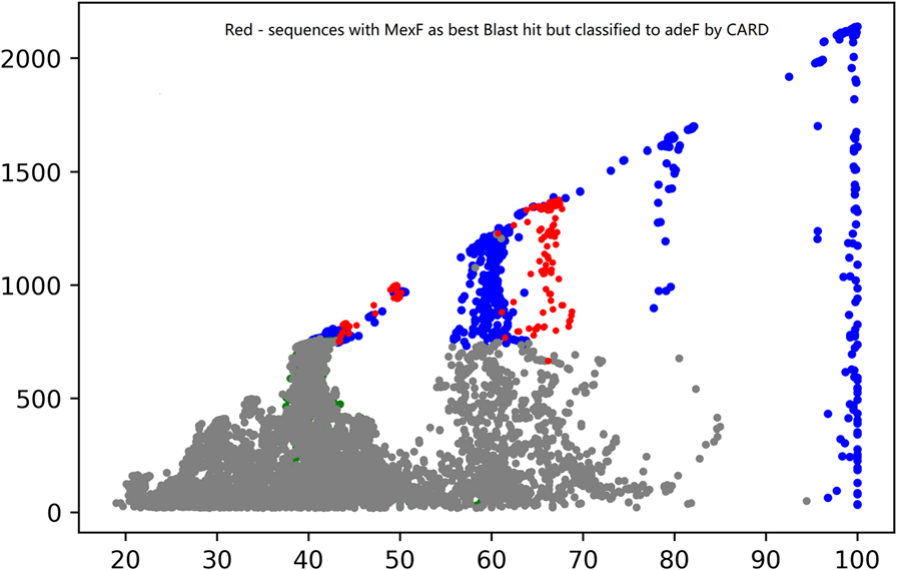
Sequences with MexF as best BLAST hits but classified to adeF by CARD

Since BLAST is the most recognized tool for evaluating homology between sequences, it’s preferable for the ARG identification models to be more coherent with the homology relationship according to BLAST. Therefore, we will seek to annotate more sequences to its best hit ARO entry. For the above example, it means to “recolor” all or a portion of red points (currently annotated to adeF) to MexF. However, the type change may cause an increase in the number of potential False-Negative in the space of adeF, shown in Fig. 4. To reflect the trade-off between, we set our objective function to be:

4 $$ {L}_{S,A}=\sum\Big( \frac{\mid {B}_i\mid }{\mid s\mid } Coherence\left({A}_i\right)-\frac{\mid {A}_i\mid }{\mid s\mid } FN\_ ratio\left({A}_i\right)\Big) $$

where |A_i_| is the number of sequences currently annotated to A_i,_ |S| is the total number of sequences.

In the next section, we will show how we can largely elevate the coherence ratio while keeping FN-ratio in a significantly smaller scale.

### Resolve ambiguity by recoloring with support vector machine

**Fig. 5:**
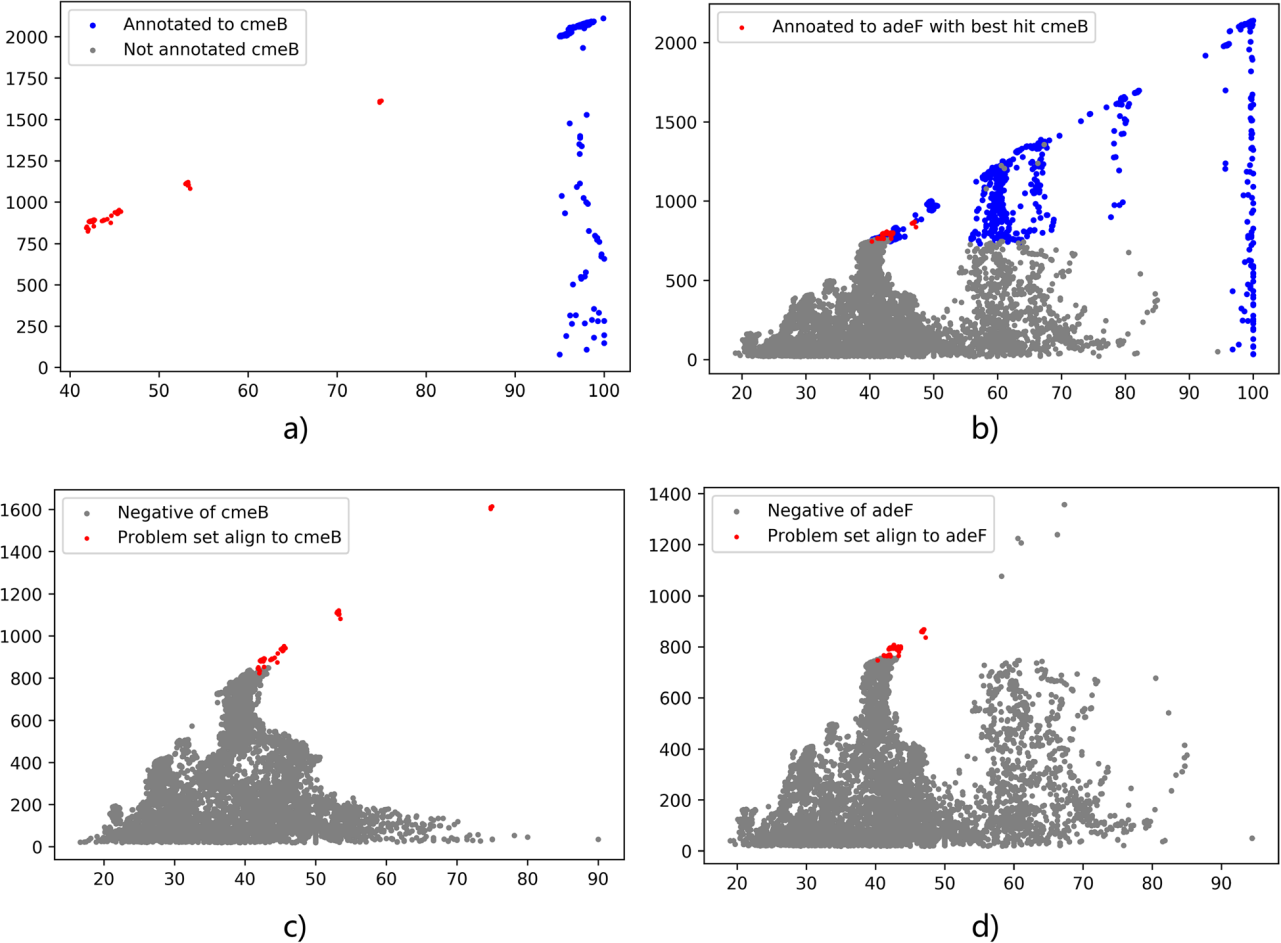
Problem Set of cmeB and their coordinates in adeF space

Given a set of query sequences S, we align them to the representative sequences of all ARO entries in CARD database. For each sequence S_i_, only the best hit with both highest bit-score and highest percent-identity is kept. If the best-hit ARO A_i_ of a query sequence is different from the ARO A_j_ assigned by the CARD model, we include this sequence to the “Problem Set” of A_i_ (denoted by PS_i_). The key point of this step is: when sequences in the problem set are aligned to two similar ARO entries, we view Align_ARO_A_i_ (S_i_) as a transform from sequence to 2D-coordinates space (Percent Identity, Bit-score). For the same set of sequences, if in the transformed space Align_ARO_A_i_ (S_i_) they are clearly distinguished with other negative hits, but in another space they are mixed together, then it’s reasonable to think that the set of sequences are true positive of A_i_ instead of A_j_. We can illustrate the argument with the problem set of ARO entry cmeB, which are annotated to adeF (Fig. 5). In the space of cmeB, the best hits of the red points are cmeB but they are annotated to adeF since bit-score cut-off of cmeB set by CARD is much higher than that of adeF. However, when we observe the problem set with the negative hits with respect to either space, we can see that in cmeB space, the problem set is clearly above negatives but in adeF space there are negatives both above the below the problem set. Therefore, it’s reasonable to say these sequences are potential false positive of adeF and true positive of cmeB.

**Fig. 6:**
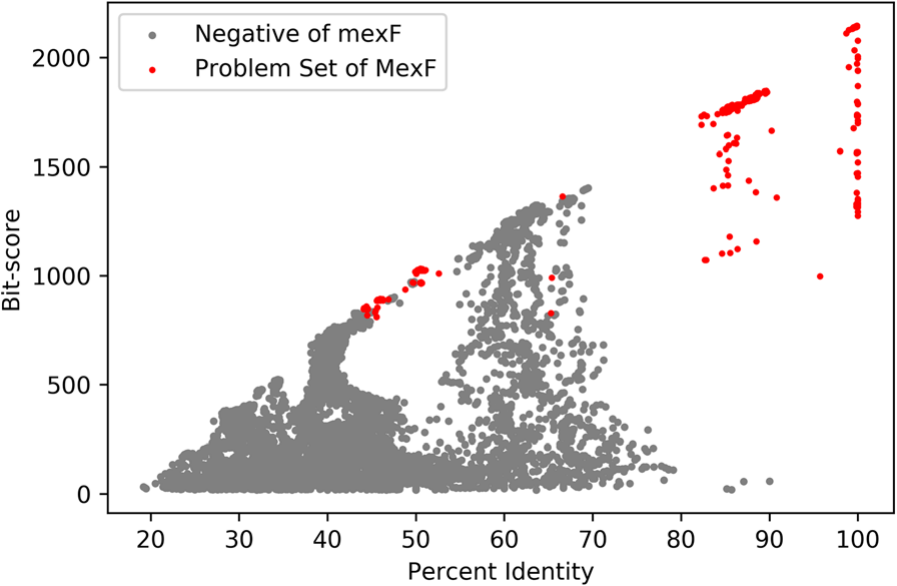
Problem set and Negatives of MexF

To quantify how far the problem set are divided with the negatives, we compute a support vector machine (SVM) in each space. The idea to use SVM is inspired by the clear linear-divisibility between a part of the problem set and the whole negatives of MexF (Fig. 6). In this situation, it’s reasonable to believe that the upper-right part of the problem set are not negatives of MexF (currently they are classified to adeF) and thus should be recoloring to MexF.

Here SVM serves as a measurement for the divisibility of points of different classes in a space. There is an established computational method for evaluating such divisibility of an SVM in python scikit-learn package, namely Platt scaling. The mean probability of the prediction on all these points can be calculated by Platt scaling. The probability computed in this way increases when the point moves away from the division line of the SVM, thus it could be used to determine which space is a better transform.

5 $$ {P}_{space}\_ ARO\left(y=1|X\right)=1/\left(1+\mathit{\exp}\left(A\ast f(x)+B\right)\right) $$

f(x) = wx + b is the division line of the SVM, and A, B are parameters trained from the prediction data by Plat scaling.

**Fig. 7:**
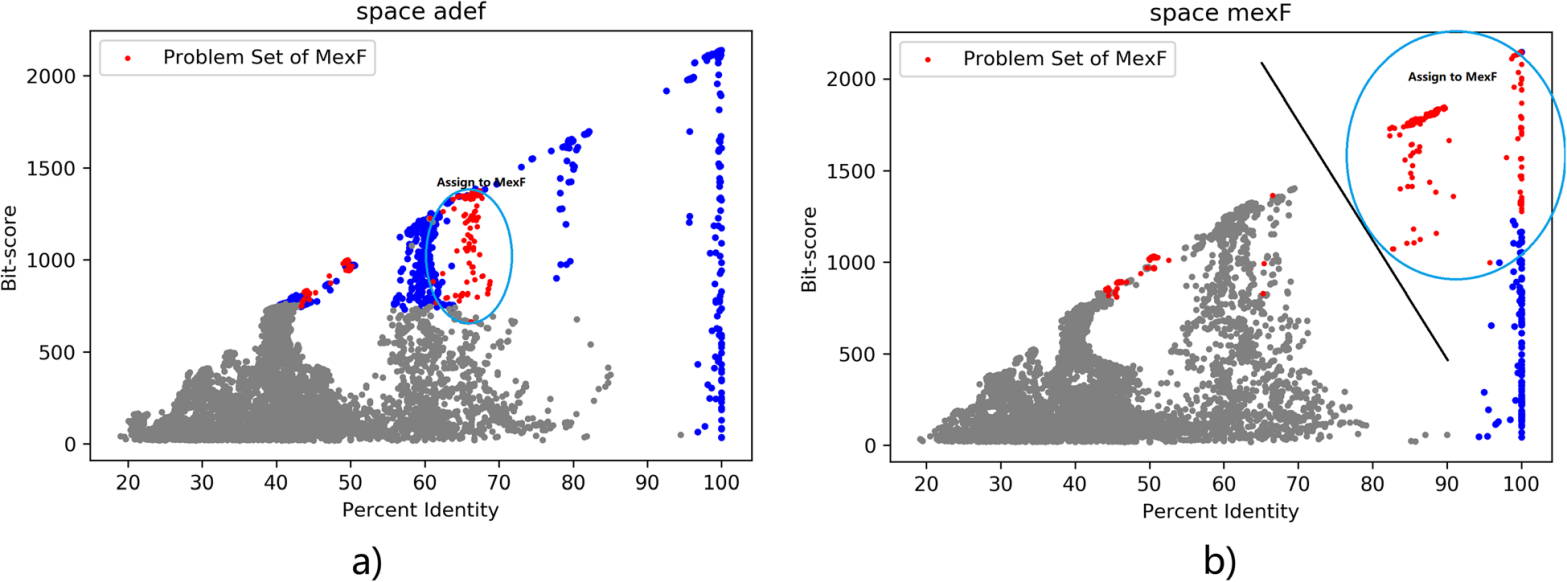
space *MexF* vs. *adeF* and recoloring

If the space of current ARO (adeF in the above case) of the problem set reports lower Platt probability, we will recolor the portion of problem set above the division line of SVM (Fig. 7) to the ARO of the best hits and update the cut-off of both AROs to their updated lowest bit-score and lowest percent identity. For cmeB where a SVM with high divisibility is computed, we use the decision function of SVM as an extra cut-off method.

**Fig. 8:**
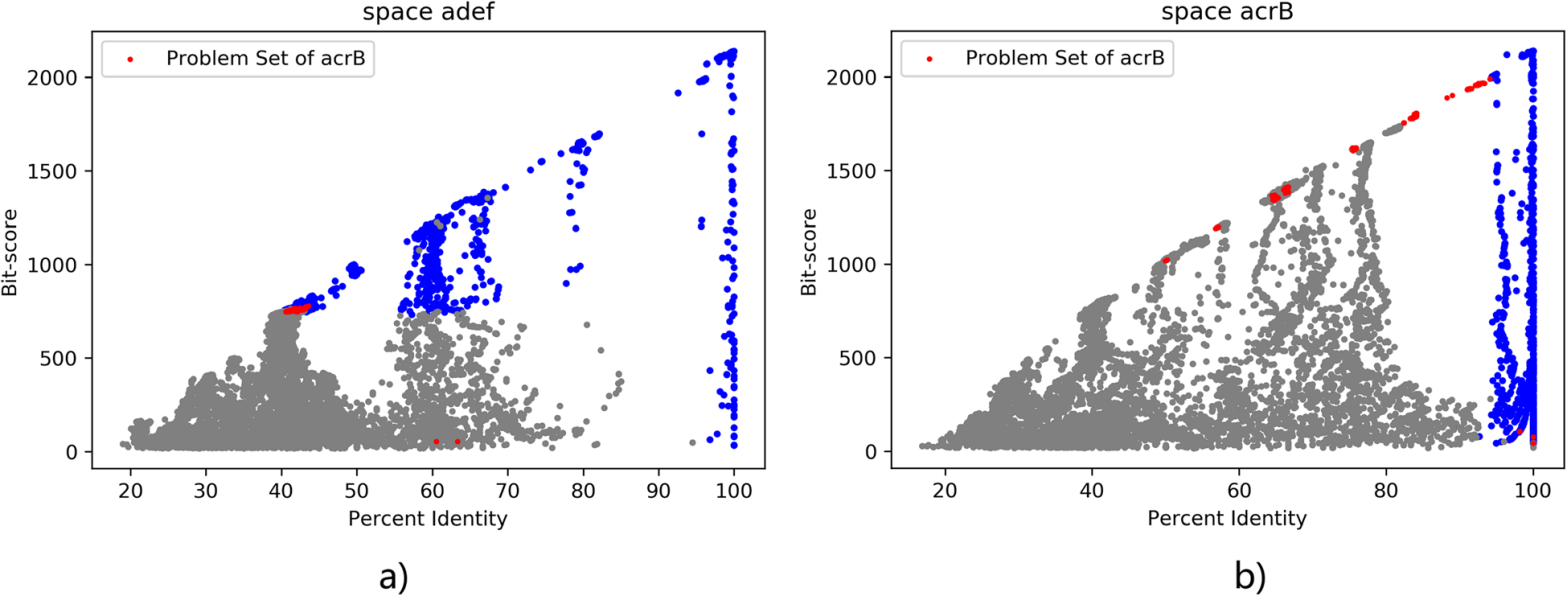
space *acrB* vs. *adeF*

Besides cmeB, there are ten other ARO entries with their problem sets containing more than 50 sequences annotated to adeF. These ARO entries are {‘ceoB’, ‘mexY’, ‘cmeB’, ‘mexQ’, ‘mdsB’, ‘oqxB’, ‘MexB’, ‘MexF’, ‘acrD’, ‘adeB’, ‘acrB’, ‘AcrF’}. We compute their problem sets, and then evaluate in which space these sequences are better divided with the negatives compared with adeF. We plot situation of *acrB* vs. *adeF* in Fig. 8. In this case, the predicted log-probability of the SVM for acrB is lower than the SVM for adeF, and we can also see from the 2d-coordinates that red points and gray points sequences in acrB displays tendency to mix with each other. Thus, we won’t consider recoloring the problem set of acrB.

In contrast, we can see a clear division between a large portion of the problem set of MexF and its negatives (Fig. 7). After computing the SVM, the right-hand portion of the problem set will be recolored to MexF.

### Formulation of categorical optimization problem

The last section demonstrated that we can increase the coherence with BLAST homology relationships while maintaining low FN ambiguous rate by “recoloring” a part of sequences. However, the above transform is more an empirical trial than a systematic optimization. Therefore, in this section we will formulate a categorical optimization problem [20] for the recoloring process between two spaces - a fixed “origin” space (adeF in our problem instance) and another “alternative space” (MexQ, MexF, etc). For a set of protein sequences G [1..N], we define a categorical variable X_i_ ∈{O, A, null (neither of the two types)} for G_i_ representing its ARG category classification. Every assignment of X[1..N] is called a “configuration”. The initial configuration is the SVM result in the last section. We have discussed that recoloring a sequence from the origin (adeF) to the alternative (MexF) may increase the coherence ratio of the alternative (MexF) space but add ambiguity to the origin (adeF) space. Suppose P of type O has higher (Percent Identity, Bit Score) than some sequences of type origin. If P is recolored to another type, then there should be a penalty to the confidence of those sequences.

For computational efficiency, we divide the Percent Identity – Bit Score map to a grid of M × N equal-sized cells. A point in the cell (x, y) in the origin space with type A will impose one unit of penalty to all points of type O in its left-down region excluding the cell itself. Let n_o_ be the number of type O points in the rectangle (0,0,x-1,y-1), N_O_ (N_A_) be the total number of type O(A) points. For each type A point (x,y) we have:

6 $$ Penalt{y}_O\left(\mathrm{x},\mathrm{y}\right)={n}_o\left(x-1,y-1\right) $$

Penalties of all type A points are added and normalized to get the total penalty on the origin space:

7 $$ Penalty(O)=\sum \limits_{point\left(x,y\right)\  of\ type\ A\ }{n}_o\left(\mathrm{x}-1,y-1\right)/\left({N}_A\ast {N}_O\right) $$

To make the optimization problem more reasonable in the biological meaning, we add an extra restriction such that the alternative space remains linear-divisible, as drawn in Fig. 7. Formally speaking, we require that there exists a line *Y = aX + b* in the alternative space such that the points of type A are all above the line. We intend to compute the slope and intercept of the optimal division line w. Therefore, our final objective function to maximize is:

8 $$ f\left(a,b\right)= Coherence(A)+k\ast Penalty(O)\kern0.37em \left(\mathrm{k}>=1\right) $$

Higher coherence indicates high potential sensitivity for A while higher penalty means potential wrong classification. Therefore, we tend to give larger weights to the later term since usually we prioritize preventing false results. However, the specific value of k depends on the need of the application and also the specific ARG type that we are concerning. Here we use MexQ to set a valid range of k, and then explore the results on MexF for k in that range. The reason we choose MexQ to set the range is that it gives the highest probability calculated by Plat-scaling in the last section, which means the problem set and the negatives of MexQ are already well-divided in the space so that we can trust the initial configuration of MexQ as the answer. Therefore, k is set to be in (1100) so that the initial configuration for MexQ is optimal.

## Results

### Results of recoloring with support vector machine

**Fig. 9:**
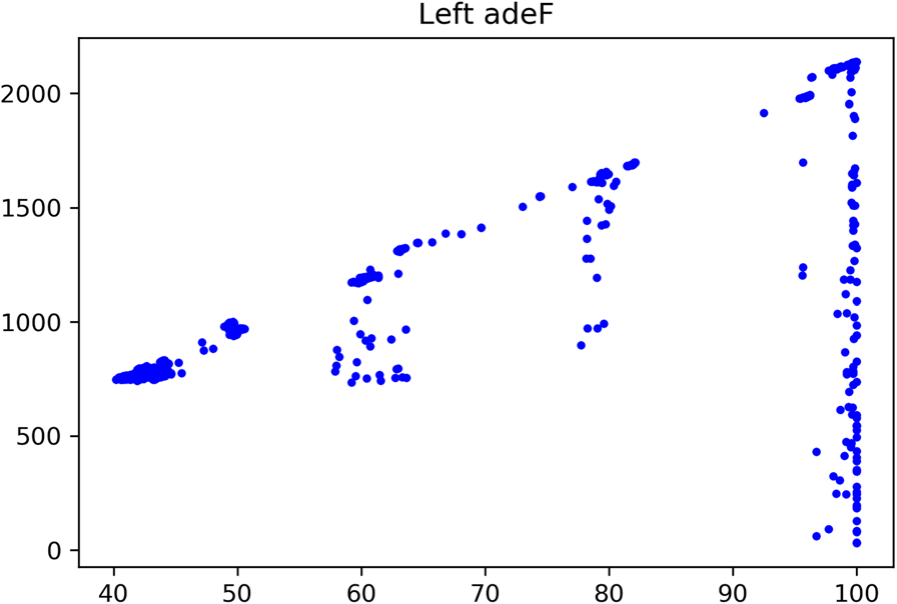
Final left sequences for adeF

**Fig. 10:**
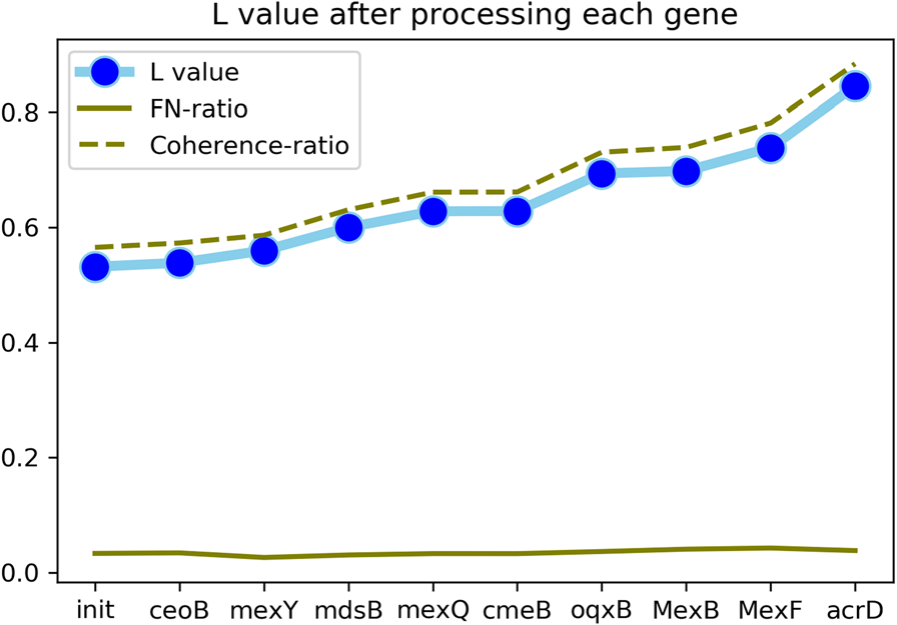
Change of L value for each recoloring

The final prevalence sequences that are classified to adeF are shown in Fig. 9 below. For the objective function *L*, the sum of coherence ratio is elevated by nearly 80% and the FN-ratio increased by less than 20%. And the coherence ratio is much larger than FN-ratio before or after. *L* value for each step of recoloring is plotted in Fig. 10. The coherence ratio rises from 56.5 to 88.4% and the FN-ratio increases little from 3.3 to 3.8%. Consequently, we increase L value from 53.1 to 84.5%.

### Results of solving categorical optimization problem

**Fig. 11:**
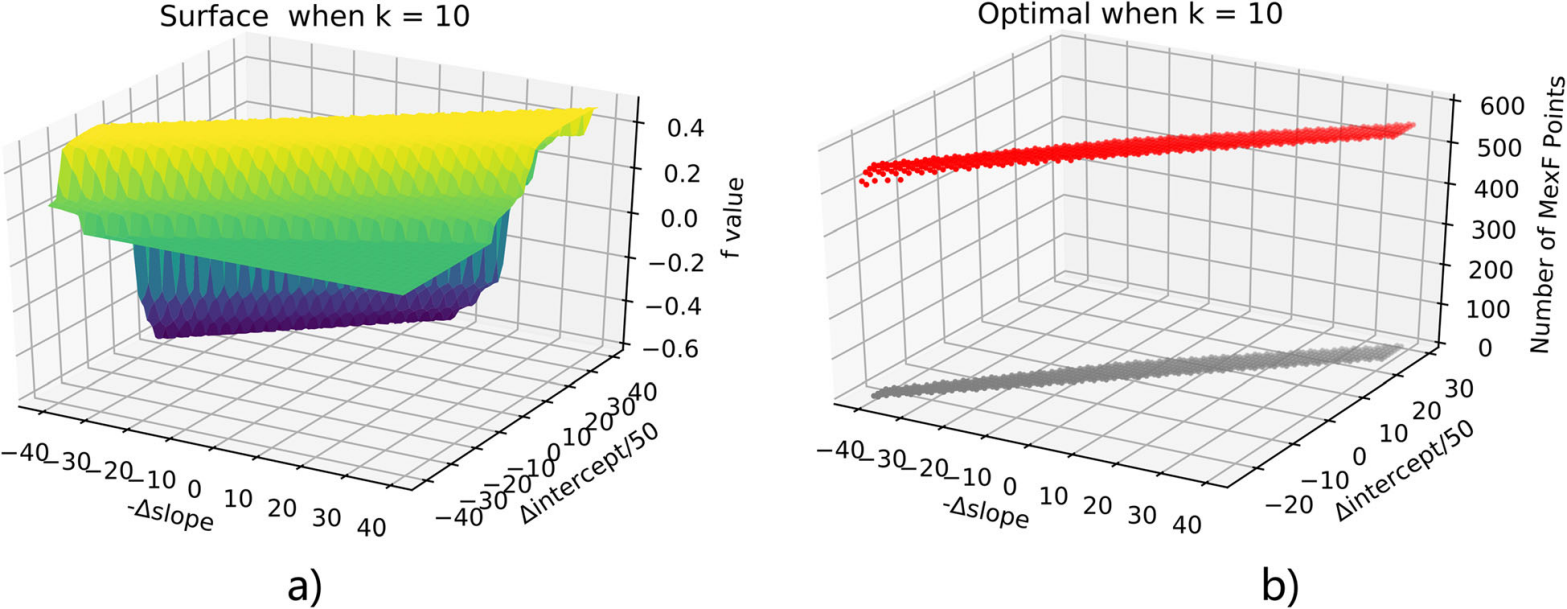
Optimization when k = 10

**Fig. 12:**
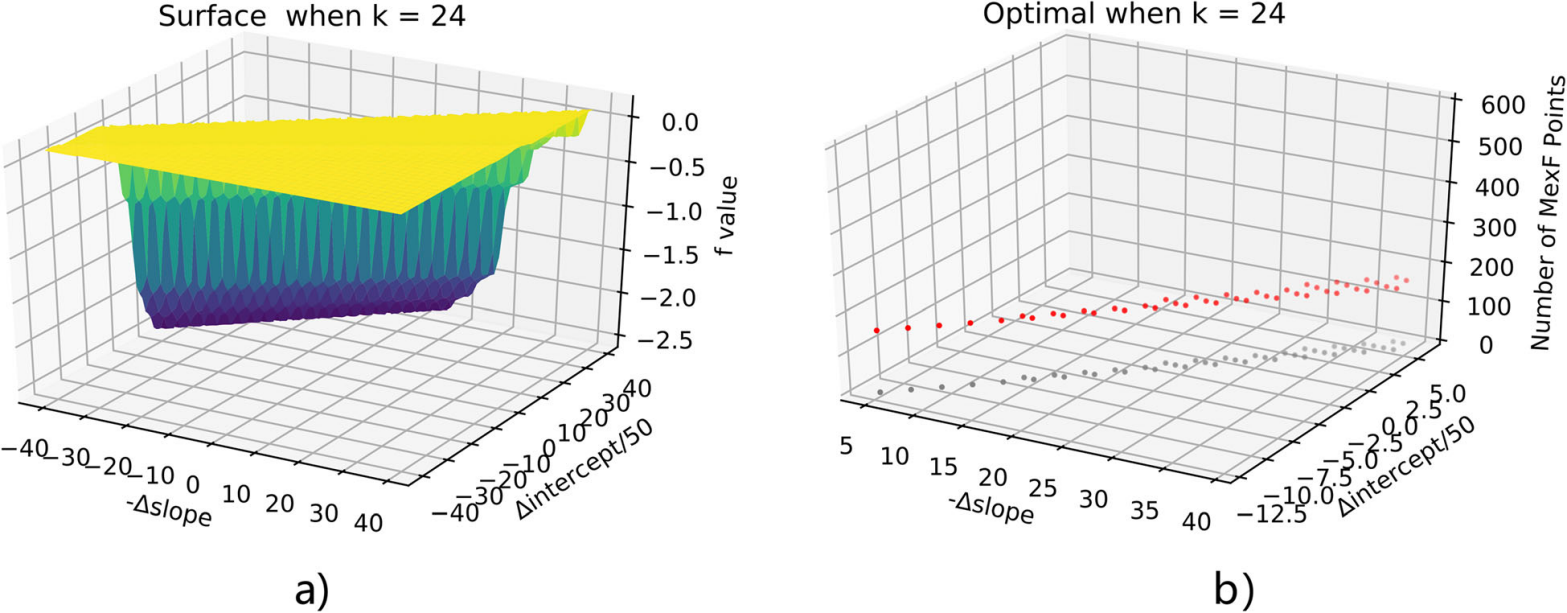
Optimization when k = 24

**Fig. 13:**
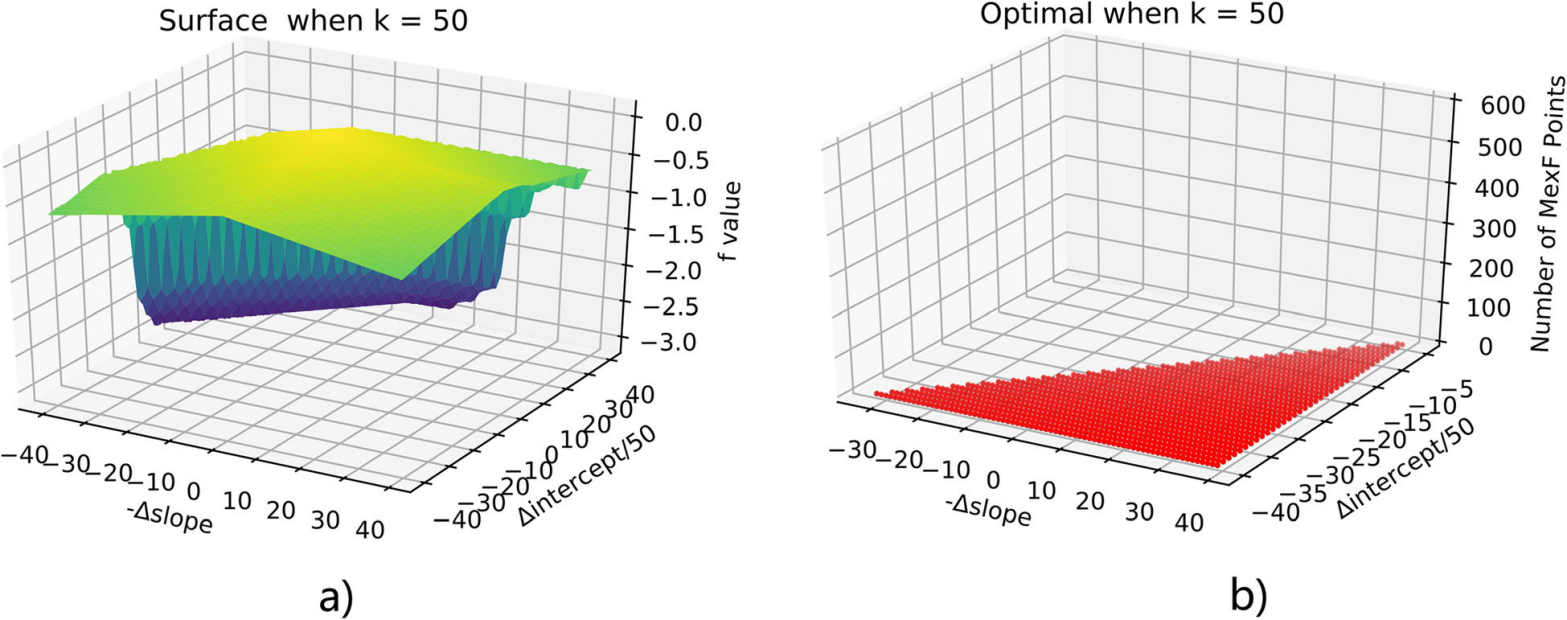
Optimization when k = 50

To solve the optimization problem, we simply apply the Monte-Carlo exploration of neighborhood configurations by randomly adjusting the slope and the intercept. By the SVM process in the last section, we have initial (slope_0_, intercept_0_) = (− 71.7, 6947.5). Exploring the optimal configurations for MexF under k values in (1100), we discover that the k = 24 as the boundary for extremely different behaviors. When k is larger than 24, the penalty term always outweighs the coherence, therefore the result is the less MexF points the better. When k does not exceed 24, the optimal points tend to fall along a line. This will be illustrated by plotting f(a,b) for k = 10,24,50 in Figs. 11, 12, 13. Notice that in all these figures (x,y) denotes the point at (slope_0_ - x, intercept_0_ + 50y) and up-left means more points assigned to MexF. For b) parts of these 3 figures, red stands for optimal points and gray are their projection onto the ground to show their coordinates clearly. We see that k = 10 and k = 24 behave similarly through optimal points when k = 10 have more MexF points.

## Discussions

CARD is a comprehensive antibiotic resistance database that provides over 2000 types of ARG entries organized in a hierarchy of gene ontology terms, together with the corresponding resisted drug classes. Moreover, the model curated from published literatures for each type of ARGs and the prevalence data are now a precious resource for researchers to know about the degree of variation among sequences in the ARG types of interest. Although we have not settled a reliable benchmark to verify the precision of an ARG discovery method, it’s already important to discover that some ARG types contain a very similar set of sequences while some others do not behave in the same way. Moreover, through in-depth analysis of approaches and effects of the classification model used by CARD, we discover ambiguous cases when sequences are aligned to ARGs of closely related types, namely RND efflux pump superfamily, which is a family of ARGs with crucial clinical importance. The decision model of CARD only considers one target ARG type at a time, which causes frequent occurrences of incoherence with BLAST homology relationships.

Through an empirical recoloring experiment, we discover that by adjusting the linear decision function of some ARG types, the occurrences of ambiguity could be reduced. And then such repairment process is formulated as a categorical optimization problem, where randomized algorithms are applied to obtain the solution to minimize the probability of ambiguous cases. Currently the formulation is limited to solve optimization of just two spaces. We look forward to developing models and solutions to consider multiple ARG types in the same family.

## Conclusions

In this article, we reviewed state-of-art antibiotic resistance gene databases and revealed ambiguous cases from the decision model of CARD. After formally defining metrics to quantify the ambiguity within CARD database, we propose recoloring with support vector machine and categorical optimization techniques to resolve the problem.

The method we raised is currently a prototype and subject to verification of more data with high-confidence annotation. Due to the rapid mutation of the pathogen genome, novel antibiotic resistance genes are keeping submitted by entities all over the globe. Therefore, the answer to our problem is deemed to be continuously dynamic. We look forward that this article can serve as a meaningful step to a systematic approach for comparing, filtering and integrating ARG reports across different reference databases.

## Data Availability

The data used in this article come from the following public available antibiotic resistance gene databases: 1. ARGs and prevalence data in CARD database, https://card.mcmaster.ca/download/0/broadstreet-v3.0.2.tar.gz [6]. 2. Resfinder ARG database, https://bitbucket.org/genomicepidemiology/resfinder_db.git/ [17]. 3. SARG database: https://galaxyproject.org/use/args-oap/ [8].

## Abbreviations

ARG: Antibiotic resistance gene
ARO: Antibiotic resistance ontology (an entry of ARG in CARD)
BLAST: Basic Local Alignment Search Tool
CARD: Comprehensive Antibiotic Resistance Database
RND: Resistance-nodulation-cell division
SARG: Structured ARG reference database
SVM: Support vector machine
